# Students’ view on supportive co-teaching in medical sciences: a systematic review

**DOI:** 10.1186/s12909-021-02958-4

**Published:** 2021-10-06

**Authors:** Afsaneh Dehnad, Maryam Jalali, Saeed Shahabi, Parviz Mojgani, Shoaleh Bigdeli

**Affiliations:** 1 English Language Department, School of Health Management and Information Sciences, Department of Medical Education, Center for Educational Research in Medical Sciences (CERMS), Iran University of Medical Sciences, Tehran, Iran; 2 Rehabilitation Research Center, Iran University of Medical Sciences, Tehran, Iran; 3 Department of Orthotics and Prosthetics, School of Rehabilitation Sciences, Iran University of Medical Sciences, Tehran, Iran; 4 Health Policy Research Center, Institute of Health, Shiraz University of Medical Sciences, Shiraz, Iran; 5 Iran-Helal Institute of Applied Sciences and Technology, Tehran, Iran; 6 Research Center for Emergency and Disaster Resilience, Red Crescent Society of the Islamic Republic of Iran, Tehran, Iran; 7 Center for Educational Research in Medical Sciences (CERMS), Department of Medical Education, School of Medicine, Iran University of Medical Sciences, Tehran, Iran

**Keywords:** Co-teaching, Students’ satisfaction, Perception, Attitude, View, Medical sciences

## Abstract

**Background:**

Supportive co-teaching (SCT) is the practice of employing two or more experts whose knowledge and experiences are needed simultaneously to make a connection across different disciplines in a classroom. Although this interdisciplinary approach seems to be beneficial, there are many features which need further examination. This study was conducted to systematically review studies addressing the use of this approach and learners’ views on SCT in medical sciences.

**Methods:**

We searched for the studies addressing students’ views on SCT in medical sciences from January1^st^ 2000 to June 31st, 2019. All the studies, both quantitative and qualitative published in English language, investigating the students’ views on SCT, in non-clinical courses in the setting of medical sciences were included. We searched electronic databases of PubMed, Scopus, Embase, Web of Science, WHO Global Health Library, Health Systems Evidence, and ERIC with the keywords and phrases related to the topic which were: “co-teaching”, “team teaching”, “collaborative teaching”, “peer-to-peer co-teaching”, “partnership teaching”, and“ teacher collaboration”.

**Results:**

By the initial search, 9806 studies were found and after deletion of duplicates and screening, 111 remained for selection. Upon the independent review by two researchers, we were able to discern 12 studies eligible to be included for data extraction. All the studies reported positive views of the students towards SCT although some identified concerns and drawbacks. The students stated that they could better perceive the relationship between basic and clinical sciences, were more engaged in the learning process, and their learning experience was optimized in a course directed by SCT.

**Conclusion:**

Overall, the students showed positive views of this approach of teaching, and their grades indicated they learned better than expected. However, mismatch and lack of coordination between instructors would make the class distracting, confusing and even disturbing. Further studies investigating different variables related to teachers and students in SCT classes are suggested.

## Background

Supportive co-teaching (SCT) is an effective teaching approach by which the knowledge and expertise of two or more teachers are employed to promote the learning process [1]. In higher education, it is recommended to apply SCT in order to benefit from the instructors’ professional knowledge, expertise, and perceptions simultaneously, and to meet the diverse needs of learners in a classroom [2]. With this approach, instructors meet before the class and perform classroom preparation including selecting, organizing, and compiling materials, setting time management, and developing lesson plans, to attend the class at the same time [3]. During the class hour(s), instructors also complement each others’ knowledge, thereby providing more comprehensive coverage of the topic, while students are exposed to the competency, knowledge, and expertise of both instructors [1].

Co-teaching is based on the social theory of Bourdieu, suggesting that human behaviors are fundamentally cultural and are formed under the influences of socio-cultural factors. Inspired by Bourdieu’s key concepts about culture, Roth and Tobin discuss that co-teaching supports the assumption that all participants sharing a classroom experience, are collectively responsible for the improvement of learning, and construct the process of learning improvement by maintaining a collaborative culture and interaction [4].

SCT represents a collaborative culture which is formed through working together, showing trust, openness, support and helping in everyday activities; with respect to SCT, educational departments work closely, teachers learn from teachers in other subject departments, thereby establishing a warm and respectful relationship, leading to shared values through which teachers’ practice and students’ learning are improved [5].

Theoretically speaking, co-teaching reflects social constructivist learning theories which focuses on social learning and interaction among participants. In this regard, Vygotsky [6] posited that meaning is constructed in social processes through discussion and group interaction; meaning that learning is a socially constructed experience, in which mental functioning is possible through interaction with others. Vygotsky maintained that meaning is constructed from discussion among group members and learning takes place through observation and social engagement with others. According to Bandura, learning takes place through modeling and observing by learners who function as active agents when they are self-motivated [7]. Co-teaching also reflects an integrated learning environment which fosters learning via synchronized retrieval of different domains of knowledge represented by the two instructors. This mental activity provides the opportunity for the learners to use integrated encoding skills which are necessary for retrieving the information, and reconstructing the memory, needed in the real world situations. In fact, when learners are exposed to separate units of information, they can integrate them by using schemas and form a meaningful chunk of coherent information, developing a basis for practicing higher cognitive skills as well [8].

Some researchers believe that integrated learning may lead to the situation in which cognitive overload occurs to the learners and is not necessarily advantageous [9]. They reason that when learners are exposed to 2 units of new information, they may not be able to process both at once. On the other hand, it is believed that learners with a prior knowledge of the subject could relate new units of information to the previously acquired schemata. However, the extent to which learners may benefit from an integrated learning environment depends on learners’ working memory capacity to aggregate information from two separate realms of knowledge and change it into a coherent mental schema [10]. Thus, the level of prior knowledge is important for integrating different types of knowledge [8].

SCT could be very demanding, as both instructors should attend the class with full coordination and preparation, which is in fact time consuming. Provision of appropriate materials, time management, and personality match between the two instructors are some of the challenges of this approach. Some instructors may not accept their co-teachers to support them because it might show they are not competent in some areas.

In medical education, SCT has been introduced as a strategy to combine theory and practice for medical students, and the literature shows that this approach is also valuable to the students of medicine [11]. The evaluation of the effect of co-teaching on learning process of medical students has shown that those medical students taught by pharmacologists and microbiologists employing SCT outperformed their counterparts at the knowledge level [12]. Willey et al. (2018) compared a class in which a microbiologist and a specialist of infectious diseases used SCT with a conventional teaching approach. Findings of their study showed that 92% of the leaners had a better understanding of the relationship between basic sciences and clinical stage, a better perception of the practical aspect of the course content, and a higher engagement in the learning process. The results of Willy’s study also showed that the learners in SCT class outperformed their counterparts [13].

In another study, SCT was used in a course called “Ideology and collaboration in social and health care”. Crow and Smith (2003) discussed that SCT was a unique experience with high efficacy for the learners. Initially, they had the assumption that SCT would endanger their autonomy as a teacher; however, they ultimately recommended that the collaboration in teaching had a positive effect on both teachers and learners. The learners enjoyed the interdisciplinary collaboration of the two experts and the instructors reported the achievement of higher self-awareness and reflectivity as well as an entrusted relationship based on constructive feedbacks enriching the classroom environment [14].

In a mixed-method study, performed in 2014–2015, SCT was examined in an education course of nursing. One of the main objectives of the study was to address the challenges of SCT in higher education. The findings showed that participants believed that SCT helped teachers have a close collaboration, harmony, mutual respect and cooperation, promoting the quality of teaching and learning [15].

In spite of favorable findings and recommendations for using SCT, there are not several studies reporting its potential employment in higher education in general and medical education in particular; thus, we set out the present study to systematically review the studies addressing learners’ views on the use of SCT in medical field. To this end, we reviewed the results of the included studies, reporting learners’ views of supportive co-teaching. In the reviewed studies, students’ views were investigated using terms such as perception, satisfaction, attitude, and evaluation.

## Methods

This study was conducted to systematically review the studies on students’ views of SCT in teaching medical sciences from January1st 2000 to June 31st, 2019. All the studies, both quantitative and qualitative which were published in English language and investigated students’ views of teaching theoretical courses at basic sciences and clinical stages in the setting of medical sciences were included in the study.

### Search strategies

In order to find published, unpublished, and other types of studies, we searched electronic databases of PubMed, Scopus, Embase, Web of Science, WHO Global Health Library, Health Systems Evidence, and ERIC. Keywords and phrases related to the topic were “co-teaching”, “team teaching”, “collaborative teaching”, “peer-to-peer co-teaching”, “partnership teaching”, and“ teacher collaboration”. To include more keywords, we searched Medical Subject Headings (MeSH), Emtree thesaurus and free-text method, and asked the experts to include more keywords. The search strategy was initially developed from PubMed and it was then extended to other databases. The search of electronic databases was completed by searching grey literature through ProQuest, Google Scholar and formal websites of organizations and credible journals manually. Finally, the reference list of the related studies and included studies were searched manually (Table 1).

**Table 1:** The search strings and the databases searched

Databases	Search strings	Number of studies
PubMed	(co-teaching [tiab] OR “team teaching”[tiab] OR “collaborative teaching”[tiab] OR “partnership teaching”[tiab] OR “teacher collaboration”[tiab])	211
Scopus	(TITLE-ABS (co-teaching) OR TITLE-ABS(“team teaching”) OR TITLE-ABS(“collaborative teaching”) OR TITLE-ABS(“partnership teaching”) OR TITLE-ABS(“teacher collaboration”))	2183
Web of Science	(TS = (co-teaching) OR TS = (“team teaching”) OR TS = (“collaborative teaching”) OR TS = (“partnership teaching”) OR TS = (“teacher collaboration”))	1814
Embase	(co-teaching:ti,ab OR ‘team teaching’:ti,ab OR ‘collaborative teaching’:ti,ab OR ‘partnership teaching’:ti,ab OR ‘teacher collaboration’:ti,ab)	272
ProQuest	(AB,TI (co-teaching) OR AB,TI(“team teaching”) OR AB,TI(“collaborative teaching”) OR AB,TI(“partnership teaching”) OR AB,TI(“teacher collaboration”))	2596

The initial studies, found by the search strategy, were exported to Endnote software 7. Then, duplicate studies were deleted and the rest of the studies were screened on the basis of the title, abstract, and objectives of the study by two researchers (S.SH and M.J) who screened the studies independently and selected the studies according to the inclusion and exclusion criteria. Any disagreement on the screening and selecting of the studies was resolved by discussion, or consultation with the third researcher (AD), if necessary.

### Inclusion and exclusion criteria

Studies which were both qualitative and quantitative, interventional (educational), with the study population of females, males, or both sexes, with the age range of above 18, and students of medical sciences at different degrees as well as medicine were included.

Review studies (narrative, systematic, etc.), conference papers, letter to the editors, studies with study population of elementary, guidance and high schools, in the setting of clinical courses, with insufficient data, published in languages other than English, and those for which full texts were not available were excluded from the review.

### Data extraction

On the basis of the study objectives, data extraction was performed, with the use of a data extraction form including: 1) name of first the author, 2) publication year, 3) country, 4) research type, 5) study’s features (discipline, course type, and degree level), and 6) main findings by three researchers (S.SH, M. J, P.M.) independently. Similar to the screening and selecting stages, any disagreement was resolved by discussion or consultation with the other researcher (A.D).

### Quality assessment

**Fig. 1:**
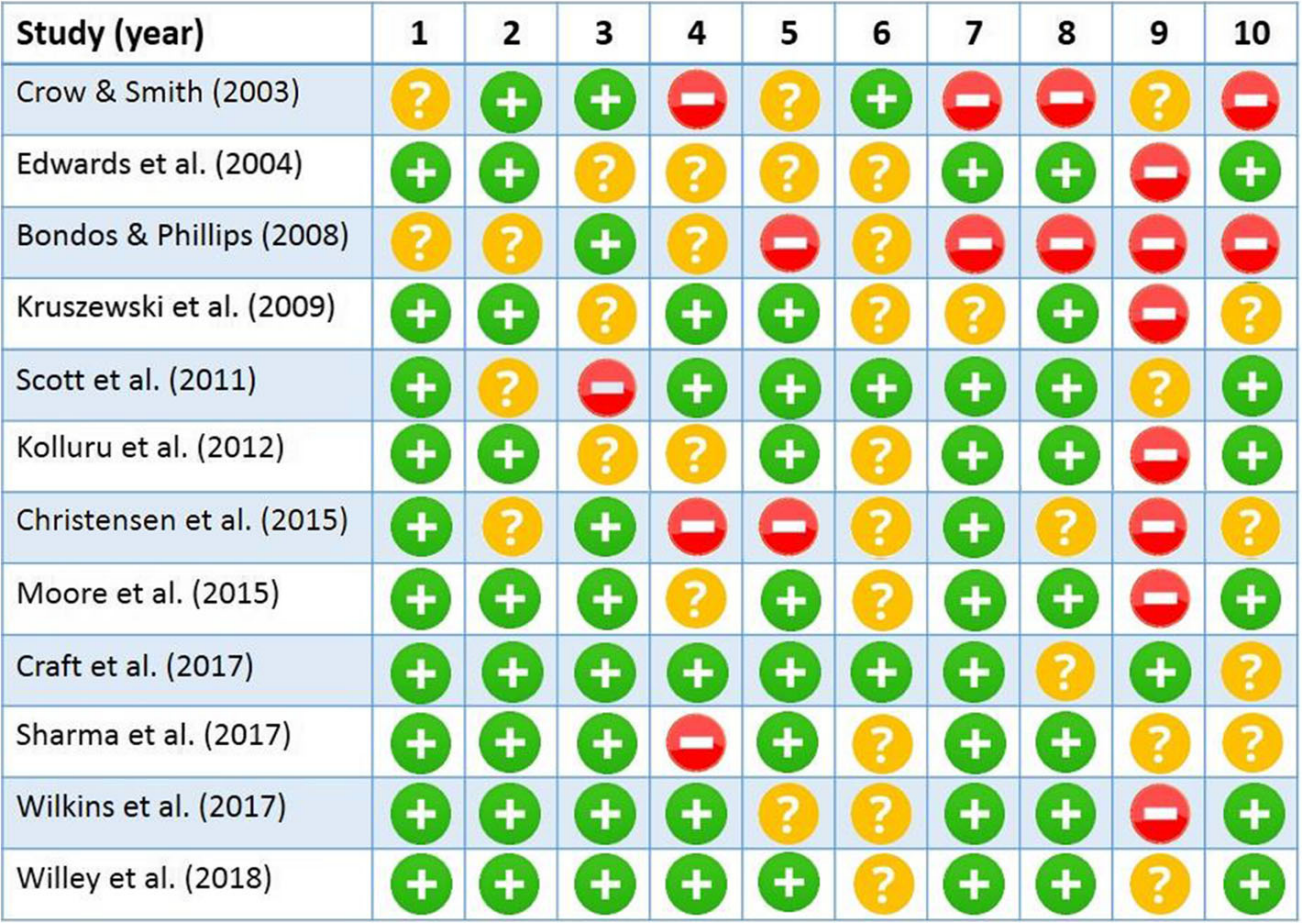
Quality assessment of the studies. Checklist for assessing the quality of quantitative studies. 1 Question / objective sufficiently described?, 2 Study design evident and appropriate?, 3 Method of subject/comparison group selection *or* source of information/input variables described and appropriate?, 4 Subject (and comparison group, if applicable) characteristics sufficiently described?, 5 Sample size appropriate?, 6 Analytic methods described/justified and appropriate?, 7 Some estimate of variance is reported for the main results?, 8 Controlled for confounding?, 9 Results reported in sufficient detail?, 10 Conclusions supported by the results?. Checklist for assessing the quality of qualitative studies. 1 Question / objective sufficiently described?, 2 Study design evident and appropriate?, 3 Context for the study clear? 4 Connection to a theoretical framework / wider body of knowledge?, 5 Sampling strategy described, relevant and justified?, 6 Data collection methods clearly described and systematic?, 7 Data analysis clearly described and systematic?, 8 Use of verification procedure(s) to establish credibility?, 9 Conclusions supported by the results?, 10 Reflexivity of the account?

Two researchers (S.SH and M.J) independently performed the quality assessment of the methodology of the included studies (Fig. 1). Any disagreement was resolved by discussion or consultation with the third researcher (A.D). The checklist of Kmet et al. [16] was used for quality assessment of both quantitative and qualitative studies. The checklist consists of 10 items for qualitative and 13 items for quantitative studies. Given that there was no input blinding in quantitative studies, the items number 5, 6, and 7 of the quantitative part were deleted and ultimately both types of studies were assessed on the basis of 10 items proposed. However, no study was removed due to a low score of quality assessment. It is noteworthy to mention that the quality assessment of the initial studies was not critical as we assumed that each study could provide us with valuable information and perspectives about the effects and aspects of SCT [17, 18].

## Results

### Synthesis and analysis of the data

**Fig. 2:**
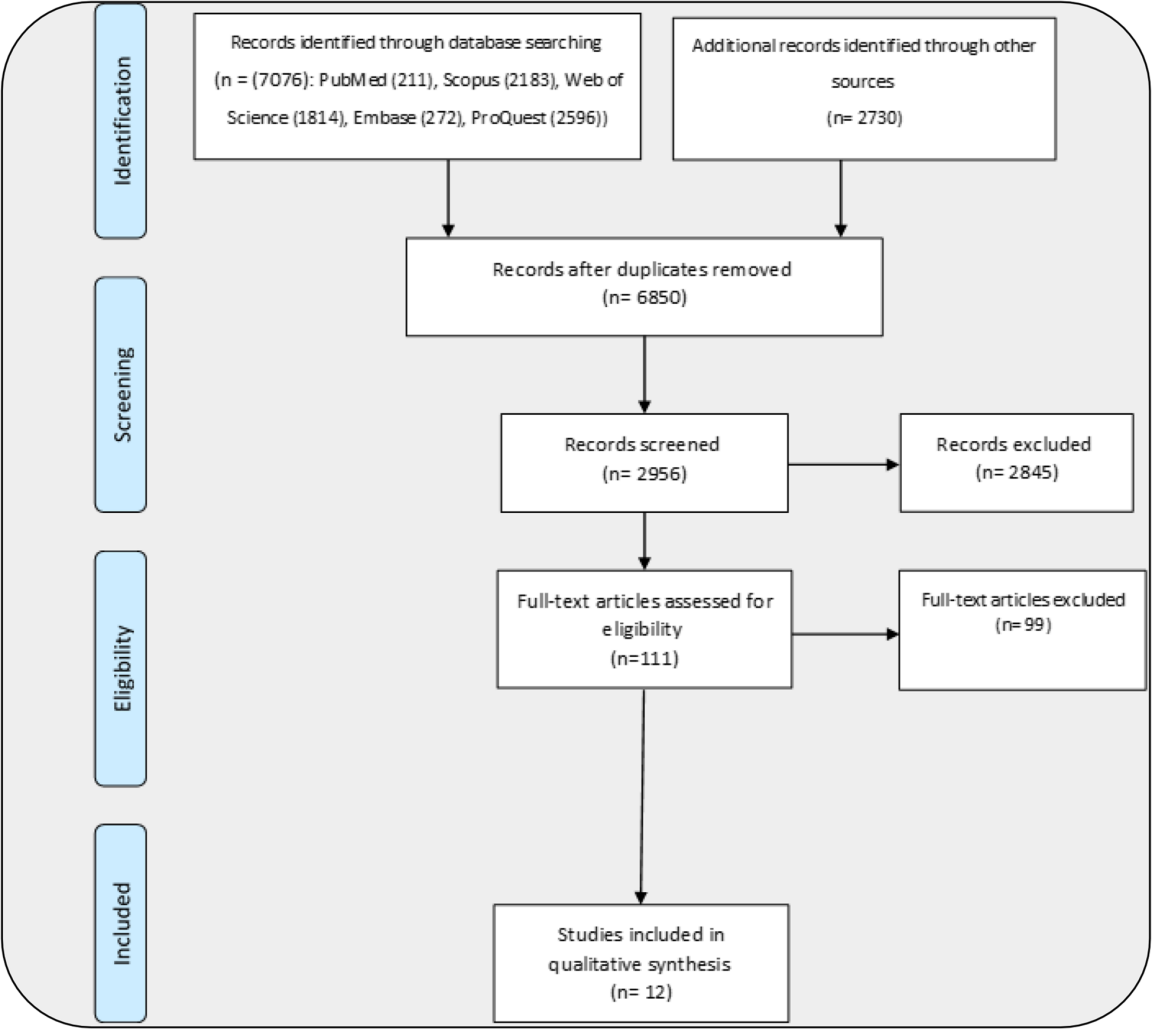
The process of searching, screening and selecting studies

Upon data extraction from each study, full texts of the included studies were reviewed and results, and discussion sections were more closely examined to have a comprehensive, and deeper perception for data analysis which was carried out via narrative analysis. The findings of the present study are reported according to the items of Preferred Reporting Items for Systematic Reviews and Meta-Analyses (PRISMA). The flowchart in Fig. 2 shows the process of searching databases, screening and final selection of studies for the review. After the primary search of databases, and other sources for published/unpublished and grey literature, 9806 studies were found. Following the deletion of duplicates, 6850 studies remained for screening which was performed on the basis of titles and abstracts, resulting in the deletion of 2845 studies. Ultimately, 111 studies remained for the selection phase, and were included for further evaluation based on full text review. Upon the independent review by two researchers (S.SH and M.J), 12 studies were eligible to be included for data extraction. Notably, there were 12 journals publishing related studies. As Table 2 shows six journals out of 12 were published in the area of specialized education. In the following section, we will report the main findings of the review.

**Table 2:** List of journals

	Study	Journal
1	Kolluro et al. 2012 [19]	American Journal of Pharmaceutical Education
2	Scott et al. 2011 [20]	Journal of Allied Health
3	Wilkins et al. 2017 [21]	Academic Psychiatry
4	Craft et al. 2017 [22]	Nurse Education Today
5	Christensen et al. 2015 [23]	Journal of Clinical Nursing
6	Sharma et al. 2017 [24]	Journal of the National Medical Association
7	Willey et al. 2018 [13]	Advances in Medical Education and Practice
8	Bondos et al. 2008 [12]	Biochemistry and Molecular Biology Education
9	Crow et al. 2003 [14]	Journal of Interprofessional Care
10	Edwards et al. 2004 [2]	Journal of Veterinary Medical Education
11	Kruszewski 2009 [25]	Journal of Nursing Education
12	Moore et al. 2016 [26]	Journal of Evidence-Informed Social Work

### Distribution of the studies reviewing SCT by discipline, year and country

In the review of the 12 studies included, SCT was investigated in the fields of pharmacology [19], medicine [2, 21, 24], nursing [22, 23, 25], rehabilitation [20], biology [12], health and social care [14], occupational therapy, and physiotherapy. Among those fields, SCT was employed in medicine and nursing more than other fields. In the time period of our search, the first study on SCT appeared in 2003, and the most recent article was published in 2018. Moreover, it was found that the highest number of publication on SCT belonged to the USA (Table 3).

**Table 3:** Characteristics of included studies

	Authors/journal	Study’s features				
		Discipline	Course	Degree	Research Type	Major Findings
**1**	Crow & Smith 2003 *Journal of Interprofessional Care* [14]	Health and social care	Ideology and collaboration in health and social care	Undergraduate	Qualitative	▪ The deliberate use of the interactions made possible by co-teaching enabled the tutors to create an active learning environment that facilitated the teaching of collaboration. ▪ Co-teaching was found rewarding and enjoyable. It is also time-consuming and costly. ▪ Co-teaching enhances student learning and improves the effectiveness of teaching. It has enormous potential for teaching interprofessional collaboration and staff development.
**2**	Edwards JC, van Walsum K, Sanders CW, Fossum TV, Sadoski M, Bramson R, and Wiprud RM. 2004 *Journal of Veterinary Medical Education* [2]	Medicine	Laparoscopy surgery	Medical and veterinary medical students	Quantitative	▪ Increase of surgical confidence, inter-professional collaboration and collaboration with veterinary students was reported by medical students. ▪ The attitudes of the veterinary medical students remained constant for the scales measuring confidence in surgical skill and collaboration with medical students. However, after the experiment, the veterinary medical students’ attitude toward inter-professional collaboration in general was significantly less positive than before.
**3**	Bondos & Phillips 2008 *Biochemistry and Molecular Biology Education* [12]	Biology	Biology	Undergraduate	Quantitative	▪ The majority of students found the course very interesting. ▪ The students were comfortable with the team-teaching approach and indicated that they would recommend the class to a friend. ▪ The course stimulated a long-term interest in biology.
**4**	Kruszewski A, Brough E, Killeen MB 2009 *Journal of Nursing Education* [25]	Second-degree nursing	Evidence-based practice (EBP) and acute care of patients and families across the life span	Bachelor of science	Qualitative-quantitative	▪ Students reported a high level of satisfaction with the shared clinical project. ▪ Students rated themselves highest on their ability to develop a clinical problem statement; retrieve, critique, and synthesize evidence; and write an EBP protocol. ▪ Students rated themselves lower in their ability to implement a practice change. ▪ Students reported they liked the opportunity to put class material immediately into practice through their EBP project.
**5**	Scott PJ, Altenburger PA, Kean J 2011 *Journal of Allied Health* [20]	Occupational and physical therapy	Evidenced based practice	Master of science for Occupational therapy students and Doctorate for physiotherapy students	Survey-qualitative	▪ Placing students with clinicians in a therapeutic environment instead of a classroom added a level of realism that further enhances student motivation. ▪ Majority of students strongly agreed with description of the course as outstanding, and believed that it stimulated thinking, and they learned a lot during the course. ▪ 25% of students reported that the real-life clinical nature of this collaborative approach was valuable for learning, and 19% felt greater motivation to participate in the project because of its potential impact on clinical practice. ▪ Surveys of the clinicians at the end of the semester revealed that most therapists did not make any change in their search for evidence.
**6**	Kolluru S, Roesch DM, Akhtar de la Fuente A 2012 American *Journal of Pharmaceutical Education* [19]	Doctor of pharmacy curriculum	Integrated pharmaco-therapy	Doctorate	Qualitative-quantitative	▪ The integrated exercise (with multiple instructors) improved perceptions of students about various aspects of the topic. ▪ Multi-disciplinary team-teaching enhanced students’ understanding of basic science concepts. ▪ Although several survey statements indicated improved student perceptions of the course compared with those of the previous year, none of the differences were significant
**7**	Christensen et al. 2015 *Journal of Clinical Nursing* [23]	Nursing	Bioscience-clinical nursing	Undergraduate	Quantitative	▪ Students indicated that they could contextualize bioscience concepts with clinical nursing practice and made links with patient care. ▪ In a nursing practice subject, a bioscientist focused on the needs of nursing students complements the nursing lecture. ▪ Second-year students indicated that they better recognized relevance of bioscience to nursing.
**8**	Moore RM, Darby KH, Blake ME 2016 *Journal of Evidence-Informed Social Work* [26]	Social work	Capstone course	Master of science	Exploratory study-Survey	▪ The more a student embraced the course content, the more confident he/she became in preparing for the comprehensive exam. ▪ All involved (faculty and students) discovered a real sense of empowerment. ▪ The decrease in the failure rate for the comprehensive exam between 2013 (18%) and 2014 (4.3%) could in part be attributed to what faculty learned from each other and how what they learned informed their teaching.
**9**	Craft et al. 2017 *Nurse Education Today* [22]	Nursing	Pathophysiology workshop	Undergraduate	Mixed methods	▪ Team-teaching that employs active learning strategies is an effective approach to assist nursing students to integrate bioscience knowledge into their nursing practice.
**10**	Sharma S, kirti R, Ali A, Takhelmayum R, Mahto M, Nair R 2017 *Journal of the National Medical Association* [24]	Medicine	Diabetes mellitus and alcohol and liver disease	Undergraduate medical student	Quantitative	▪ Both conventional and co-teaching were significantly effective in increasing the knowledge scores (*p* = 0.0001) with no significant difference (*p* = 0.59) in learning outcomes between the two. ▪ Co-teaching showed better knowledge retention compared to conventional teaching (*p* = 0.008).
**11**	Wilkins KM, Moore D, Rohrbaugh RM, Briscoe GW 2017 *Acad Psychiatry* [21]	Medicine	Dementia conference	Undergraduate	Quantitative	▪ Students in the flipped curriculum group were significantly more likely to report that the session enabled them to achieve a deeper understanding of the clinical science of dementia compared to participants in the co-teaching group. Other survey responses did not differ significantly between the two groups. ▪ Both groups agreed that the integration of basic and clinical science (as it was presented in their respective session format) should be implemented elsewhere in the curriculum
**12**	Willey JM, Lim YS, Kwiatkowski T 2018 *Advances in medical education and practice* [13]	Medicine	Immunology/microbiology course	Undergraduate and graduate	Quantitative-qualitative analyzed for themes.	▪ A significant majority of students (92%) reported they understood the connection between basic and clinical sciences better with co-teaching (compared to solo-teaching). ▪ A plurality of students indicated that co-teaching provided a better overall learning experience (81%), was more engaging (74%) and made it easier to apply content (74%).

### Distribution of the studies by the research method

Considering the research methods of the selected studies, five studies employed mixed methods, six studies were conducted by using quantitative methods while one study used qualitative method. The terms used synonymously for SCT were “Co-teaching”, “Collaborative teaching”, “Integrated teaching”, and “Team teaching”, and the terms used as an alternative for SCT were “Conventional teaching” and “solo-teaching”.

### Distribution of studies by participants’ characteristics

With regard to characteristics of teachers, only instructors’ special fields of teaching in some studies were given, and there was no mention of job experience, age, gender, etc. in the reviewed studies. The number of participants, in the studies reviewed, ranged from 9 to 199. For students’ characteristics, disciplines, educational grades, and courses taught by SCT were mentioned in the studies. A few studies mentioned the age range of participants; however, it was 22–45 in the study by Kruszewski et al. [25], in 2009, an average of 24 in the study by Willey et al. [13] in 2018, 22–28 in the study by Yalon et al. [27], in 2017, and 20–29 and 30–39 in the study by Yoon et al. [28] in 2014. Yet, other student characteristics were not mentioned in the reviewed studies.

There were four themes emerging from the review of the included studies. These themes mainly reflect the students’ positive views which will be presented in the following section.

### Integration of theory and practice

Kolluru et al. [19] in Texas A&M examined SCT in a study conducted on an integrated curriculum of pharmacotherapy, taught by basic and clinical sciences instructors, without any control group. The students reported that co-teaching was very advantageous and they were able to meet the learning objectives of the course. Many of the students mentioned that contradictions would happen if instructors had to teach individually.

Christensen et al. [23] reported a co-teaching class offered by bioscientists and academic nurses teaching to sophomore students of nursing whose perspectives about SCT were then evaluated. On the basis of the findings, more than 90% of the respondents approved the collaboration between bioscentists and nursing instructors in teaching pharmacology and believed it facilitated learning about patients’ conditions.

Sharma et al. [24] reported a study in which co-teaching was employed on two topics of diabetes and alcohol drinking, and liver diseases in India. Both groups were taught the same subject matter but one group with integrated teaching and the other group with conventional method. Pre and post tests were carried out, and the students’ perception of teaching and learning experience was examined through a survey after the intervention. It was found that the knowledge outcome improved significantly in both groups and there was no significant difference between the two groups. On the basis of the survey, the students showed that they preferred co-teaching to conventional teaching. More than 95% of the students believed that co-teaching would provoke interest and enthusiasm for learning and help to enforce the employment of basic sciences in clinical learning situations.

SCT was explored by Willey et al. in a course of immunology/ microbiology offered at a medical school. According to the results of the study [13], the students were able to perceive the key concepts of basic sciences better when the course was taught by instructors from basic sciences and clinicians. They also reported that their overall learning was improved, they perceived the relationship between basic sciences and clinical sciences, they were more engaged in the learning process, and their overall learning experience was optimized.

In this regard, comparing flipped and co-teaching formats for integration of basic and clinical science in psychiatry clerkship, Wilkins discussed that although participants had positive view for both formats, students engagement with co-teaching was higher [21].

Craft et al. [22] in a study on nursing students concluded that co-teaching assist students to integrate knowledge into practice.

### Active learning

In the study by Crow & Smith (2003) [14], SCT was investigated in an undergraduate course of Ideology and Collaboration. The instructors collaborated on organizing materials and developing study guides for the learners. In addition, they had frequent reflection on teaching and facilitating group discussions. The learners (*N* = 31), who were asked to write their feedbacks, had positive views about SCT. One of the themes extracted from their feedbacks was the importance of interaction in the class. The students approved of the interaction between the instructors as a stimulus to trigger active participation of students who were even interested in pinpointing very delicate signs such as nonverbal interaction, suggesting the significance of the details of interaction and its perception by students. The other themes emerged were convenient classroom environment, and the equal share of each instructor for intervention as well as mutual respect between them. Sense of humor, and making use of differences between teaching approaches of instructors along with role modelling collaboration were mentioned as positive points of the approach.

Kruszewski et al. [25], 2009, reported a study in which two instructors of evidence-based practice (EBP) and acute care of patients and families was designed with a collaborative approach to teaching. The students showed high satisfaction with this approach of teaching, and their grades (ranging: 6–9.36) showed they learned better than expected.

Craft et al. [22] performed both quantitative and qualitative assessment of the effect of a team-teaching workshop on the practical knowledge of pathophysiology from the perspective of senior students of nursing. At the end of the workshop, students’ perspectives were assessed by a questionnaire consisting of 14 questions. To have a deeper understanding of the students’ attitudes, a focus group of 4 students was held and the students were interviewed by a nurse researcher for 40 min. The students reported that team teaching was a very helpful experience for the learning of that subject matter. They reasoned that a biostatistics instructor would not be able to relate the concepts of pathophysiology to professional duties of a nurse, if he/she had solo teaching. However, by the co-teaching method, it was possible to covey the concepts of pathophysiology in the context of nursing profession, to understand basic concepts, and realize how a physiological assessment could lead to a nursing intervention. The quantitative findings of the study showed that the students were able to understand the relationship between theoretical bioscience and clinical nursing.

### Real-life learning

Scott et al. [20] developed a curriculum on the basis of the collaboration between faculty members and clinicians for teaching EBP to the students of physiotherapy and master students of occupational therapy. Student satisfaction, and value perception of students from the collaboration between clinicians and faculty members were evaluated via a standard evaluation instrument. More than 88% of them believed that SCT was thought-provoking in the process of evidence-based clinical decision making. More than 70% evaluated this course very good, while more than 79% described the course to be very informative.

Moore et al. [26] offered an EBP course for master students of social workers for three universities, with a revised curriculum based on the collaboration of faculty members of the universities. The students’ rate of failure in the comprehensive exam in comparison to the last 3 years decreased significantly. The results of the evaluation indicated that employing team teaching would lead to the integration of the contents of the two different subject matters which in turn would meet the future professional expectations of the students.

### Inter-professional collaboration

SCT was examined in Texas A&M University by Edwards et al., with 50 students of medicine and 30 students of veterinary, who volunteered to take part in the study, and to be trained for surgical skills. A questionnaire, with 21 questions, surveying medical students’ attitude was administered to evaluate three dimensions of inter- professional collaboration (8 questions), confidence on surgical skills (7 questions), and team work with the students of veterinary (6 questions). A similar three dimensional questionnaires with 34 questions was given to the students of veterinary. The findings showed a significant increase in all dimensions of the students’ attitude including inter-professional collaboration, confidence in surgical skills, and collaboration with veterinary medical students [2]. Similarly, the development of the concept of inter-professional collaboration was reported in the study of Crow & Smith [14] highlighting it in their study based on their investigation of co-teaching on a group of multidisciplinary undergraduates.

### Communication skills

Bondos et al. [12] examined SCT for teaching biology to non-majors. The results of the survey showed that the students were content with SCT approach, and interested in studying biology, while an appropriate communication skill was developed.

In the same vein, the occupational therapy and physiotherapy students participating in the study of Scott et al. [20] reported that communication skills were developed by co-teaching.

Willey et al. [13] also reported that students believed co-teaching was effective in enhancing communication skills across disciplines.

### Challenges

Despite the many benefits of co-teaching, this model of teaching also faces a number of challenges. Based on the findings, discontinuity, providing repetitive contents, and existing discouraging arguments and discussion in co-teaching courses were the major drawbacks, which can lead to students’ dissatisfaction [12, 25] Moreover, friction between lecturers, according to the findings of Crow and Smith, can be a major drawback in SCT [14]. These findings indicate that if co-teaching is accompanied with unequal share of each instructor, and is based on power practice of one side without mutual respect could be hazardous. The learners mentioned that the tension between the instructors would bring a disturbing environment for both teachers and students [14].. In response to the challenges of SCT, employment of a coordinator, meeting of the instructors for planning and maintaining coordination, and documenting the duties and responsibilities of each instructor can reduce confusion and tension [14, 24].

## Discussion

SCT is described as the teaching to a single group of students by instructors from different professional or academic backgrounds [14]. In medical education this method has been used to bridge the gap between theory and practice [13, 18, 19, 21, 22, 24], to teach evidence based practice [20, 25], and in teaching programs having sensitive contents, such as breaking bad news, counselling, and ethical decision making [29]. The present study in a search to explore students’ perceived views with regard to SCT systematically reviewed the relevant studies in medical sciences. After a comprehensive search, on the basis of the inclusion criteria 12 studies were reviewed thoroughly and closely.

Earlier studies have reported advantages including higher participation, exposure to different philosophies, experiences, and sources of education in co-teaching. Anderson & Speck 1998 [30], Hatcher et al. 1996 [31]. In particular, Bakken et al. 1998 [32] suggested that co-teaching could represent an appropriate role model for students. According to the findings of the present study, more recent studies have also highlighted the advantages of SCT and have reported students’ positive views due to different reasons which will be discussed as follows.

The students’ positive view in the reviewed studies was mainly due to the integration of theory and practice which was achieved when two instructors from two disciplines collaborate to provide a convenient learning environment leading to the enhancement of effective learning [13, 19, 21–24]. This finding suggests that SCT could foster learning through synchronized retrieval of different domains of knowledge represented by the two instructors. Through this mental activity, students can practice integrated encoding skills which are required for the retrieval and integration of information in their future practice where they have to recall, integrate, and reconstruct what they have learned in theoretical and practical classes [8]. The convenient environment, interaction, and mutual respect between instructors created by SCT [14], the synchronous presence of two instructors who supported each other to convey the meaning of concepts related to different disciplines efficiently, [22], as well as the integrated learning environment engage learners to experience an enjoyable and effective learning process [13]. These findings, reflect the actualization of SLTs positing that meaning is constructed in social processes through interaction and discussion [6] and learning could take place via role modeling [7].

In addition to providing a deeper and more comprehensive understanding of the subject, SCT, can be a practice to prepare students for the challenges they might face in future professional situations. Greater perceived relevance of the subject to practice and higher motivation have also been reported [20, 23].

Scott et al. [20] and Moore [26], reported that integrated teaching led by SCT can meet the future professional expectations of learners, where they have to employ and practice the skills they learn simultaneously and in an integrated way. Indeed, SCT can prepare students for the real life situations, reflecting collaborative culture and integrated learning environment.

The students in the study of Scott [20] reported that the simultaneous presentations of the two instructors was informative and thought provoking indicating the success of inter-professional collaboration resulting in effective learning through the development of a collaborative culture. Moreover, Participants in Edward’s study [2] were satisfied with the co-teaching because the collaboration between the two instructors served as a good role model.

Many medical students will work in the fields requiring collaboration with other professional groups in their future career. In fact, multidisciplinary and interdisciplinary team approaches are often employed or developed in various fields of medical sciences. Overlaps in the professional fields of medical sciences, in addition to legal and ethical academic issues, require tolerance and cooperation between groups with common or conflicting interests. SCT can teach students that a single subject can be approached differently and addressing diverse aspects of a topic allows for a better and more integrated perception of it. In fact, learners are expected to go through the same mental process in their future careers. According to the findings of the reviewed studies, the inter-professional collaboration, provides the opportunity for students to learn about communication skills [12, 13, 20] suggesting that meaning is constructed in social processes through group interaction, observation, and modeling [6, 7].

However, joint planning and reflection are time consuming and need openness as well as developing and maintaining communication skills. Moreover, addressing two different aspects of the same thing may be distracting for students; thus instructors need to commence and proceed their instruction on the basis of the prior knowledge of students. There are concerns that the bilateral discussions and dialogues between the instructors might lead to having passive learners [33];however, fostering a collaborative culture in which all participants including teachers and learners are responsible for the process of learning can avoid this problem.

Managing any tension or friction between teachers is of great importance because even the non-verbal exchange between teachers are subjected to close scrutiny by students [14]. In Willey’s (2018) study [13] the students noticed that some sessions were disjointed or distracting because of the lack of coordination between the two instructors and mentioned that the method requires “a good dynamic” between scientists and clinicians. The coordination of instructors before the class sessions, and the personality match between instructors are crucial elements of SCT [2]. In the study of Kollura et al. [19], it was discussed that when teaching alone, contradictions on different subject matters might have happened while contradictions were avoided when the two instructors attended the class simultaneously. This finding highlights the importance of developing and maintaining a collaborative culture supported by educational administrative bodies in higher education.

As with any teaching process, instructors need to be motivated to participate, as well as having the required personality characteristics and competencies. Failure to pay attention to this issues, may lead to contradictions between instructors and confusion among students. Therefore, in addition to developing a collaborative culture and supporting the expansion of SCT, the mismatch in personality characteristics of the two instructors should not be dismissed.

There is no shadow of doubt that SCT is time consuming and needs to be well managed and moderated to outstand among other approaches. It may also be costly in some academic settings, and there may be administrative barriers in employing this approach, both of which need support of educational administrative bodies.

It is worth mentioning that in a few of the studies reviewed the employment of technology was mentioned in SCT courses. Bondos et al. [12], employed a website for providing a source of information regarding course policies, homework, assignments, study guides, tour dates and places, and lecturer contact information (online schedule). Moreover, Kolluro et al. [19] used Blackboard (Blackboard Inc., Washington, DC) for posting recorded sessions for the students to be used later. In the rest of the reviewed articles, the use of technology was not mentioned, this might be due to the fact that the studies were conducted at the time when online courses were not the main medium of instruction. However, this issue needs to be addressed in future studies.

### Strengths and limitations of the study

One of the strengths of this study is the comprehensiveness search of the electronic databases. To the best of our knowledge, this is the first systematic review on this topic in medical sciences, which can provide an appropriate ground for future studies.

However, the findings of the reviewed studies showed some drawbacks. In the selected studies, only instructors’ special fields of teaching in some studies were given, and teachers’ characteristics including years of experience, age, and gender were not mentioned while the relationship between these variables and SCT is important. Some of the studies were conducted with a small sample size and convenient sampling; thus, caution should be taken in generalizing the results. The number of quantitative research studies was higher in the selected studies which might be due to the easier assessment of the attitudes by available questionnaires. However, qualitative studies for obtaining deeper understanding of SCT are also recommended in the future studies.

The included studies did not examine similar relevant variables and methods in the evaluation of the teaching methods nor in the students’ views; therefore, making comparison of the results is difficult, and generalization should be taken with caution. Moreover, some of the studies did not have a control group, and others only performed comparison with previous studies [19]. Regarding students’ views, all the studies have reported positive attitude of the students although some have reported concerns and drawbacks. Similarly, the studies addressing SCT have also shown improvement, except one study which did not report a statistically significant difference between the conventional teaching and co-teaching [24]. Identifying the right subject for co-teaching, designing the right course plan, and appropriate assessment are the topics which need to be examined in the future studies**.** All in all, due to considerable methodological heterogeneity among the included studies, lack of sufficient quantitative data to perform meta-analysis, and lack of a comparison group in many of the included studies, the results should be taken into consideration with caution.

## Conclusion

SCT is a novel approach of teaching emerging within a collaborative culture and on the basis of the SLTs. All the studies reviewed reported positive views of the students on SCT. The students stated that SCT help them to perceive the relationship between basic and clinical sciences, they were more engaged in the learning process, the simultaneous presence of the two teacher provided them with a good model of inter-professional relationship and communication skills they would need in real life situations. However, some studies reported challenges and concerns regarding SCT, including personality mismatch and lack of coordination between instructors which would make the class distracting, confusing and even disturbing. The findings of the present study can contribute to the promotion of SCT, development of a collaborative culture, and design of innovative curriculum in which SCT has its own place. The issue regarding the challenges of SCT, the employment of technology in SCT courses, and support of educational administrative bodies need to be explored in future studies. Due to the methodological diversity of the included studies and the variables studied, it was difficult to compare the results; thus, generalization should be taken with caution.

## Data Availability

Not applicable.

## Abbreviations

SCT: Supportive co-teaching
MeSH: Medical Subject Headings
PRISMA: Preferred Reporting Items for Systematic Reviews and Meta-Analyses
EBP: Evidence-based practice

## References

[1] Bacharach N, Heck TW, Dahlberg K. Co-teaching in higher education. J College Teach Learn. 2008;5(3). 10.19030/tlc.v5i3.1298.

[2] Edwards JC, van Walsum K, Sanders CW, Fossum TV, Sadoski M, Bramson R, Wiprud RM (2004). Attitudes of veterinary medical students and medical students toward collaborative learning: an experiment. J Vet Med Educ.

[3] Ferguson J, Wilson JC (2011). The co-teaching professorship: power and expertise in the co-taught higher education classroom. Scholar Pract Quart.

[4] Roth W-M, Tobin K (2001). The implications of Coteaching/Cogenerative dialogue for teacher evaluation: learning from multiple perspectives of everyday practice. J Pers Eval Educ.

[5] Shakenova L (2017). The theoretical framework of teacher collaboration. J Hum Soc Sci.

[6] Vygotsky LS. Mind in society: the development of higher psychological processes. Massachusetts: Harvard University Press; 1978.

[7] Bandura A. Social foundations of thought and action: a social cognitive theory. Massachusetts: Prentice-Hall, Inc; 1986.

[8] Harr N, Eichler A, Renkl A (2015). Integrated learning: ways of fostering the applicability of teachers’ pedagogical and psychological knowledge. Front Psychol.

[9] Ainsworth S, Bibby P, Wood D (2002). Examining the effects of different multiple representational Systems in Learning Primary Mathematics. J Learn Sci.

[10] Kalyuga S (2008). When less is more in cognitive diagnosis : a rapid online method for diagnosing learner task-specific expertise. J Educ Psychol.

[11] Lee M, Gaard MB (2015). Co-teaching the basic sciences,does it really influence student outcomes?. FASEB J.

[12] Bondos SE, Phillips D (2008). Team-teaching a current events-based biology course for nonmajors. Biochem Mol Biol Educ.

[13] Willey JM, Lim YS, Kwiatkowski T (2018). Modeling integration: co-teaching basic and clinical sciences medicine in the classroom. Adv Med Educ Pract.

[14] Crow J, Smith L (2003). Using co-teaching as a means of facilitating interprofessional collaboration in health and social care. J Interprof Care.

[15] Lock J, Clancy T, Lisella R, Rosenau P, Ferreira C, Rainsbury J (2016). The lived experiences of instructors co-teaching in higher education. Brock Educ.

[16] Kmet LM, Lee RC, Cook LS (2004). Standard quality assessment criteria for evaluating primary research papers from a variety of fields.

[17] Whittemore R, Knafl K (2005). The integrative review: updated methodology. J Adv Nurs.

[18] Webb C, Roe B (2007). Reviewing research evidence for nursing practice: systematic reviews.

[19] Kolluru S, Roesch DM, Akhtar dela Fuente A (2012). A multi-instructor, team-based, active-learning exercise to integrate basic and clinical sciences content. Am J Pharm Educ.

[20] Scott PJ, Altenburger PA, Kean J (2011). A collaborative teaching strategy for enhancing learning of evidence-based clinical decision-making. J Allied Health.

[21] Wilkins KM, Moore D, Rohrbaugh RM, Briscoe GW (2017). Integration of basic and clinical science in the psychiatry clerkship. Acad Psychiatry.

[22] Craft J, Christensen M, Bakon S, Wirihana L (2017). Advancing student nurse knowledge of the biomedical sciences: a mixed methods study. Nurse Educ Today.

[23] Christensen M, Craft JA, Wirihana L, Gordon CJ (2015). Pathophysiology team teaching: bioscientist contribution to knowledge integration in a nursing subject. J Clin Nurs.

[24] Sharma S, Ravikirti AA, Takhelmayum R, Mahto M, Nair R (2017). Co-teaching: exploring an alternative for integrated curriculum. J Natl Med Assoc.

[25] Kruszewski A, Brough E, Killeen MB (2009). Collaborative strategies for teaching evidence-based practice in accelerated second-degree programs. J Nurs Educ.

[26] Moore RM, Darby KH, Blake ME (2016). A collaborative team teaching model for a MSW capstone course. J Evid Informed Soc Work.

[27] Yalon-Chamovitz S, Kraiem Y, Gutman C (2017). Deconstructing hierarchies: Service users as co-teachers in occupational therapy education. Work.

[28] Yoon SP, Cho SS (2014). Outcome-based self-assessment on a team-teaching subject in the medical school. Anatomy Cell Biol.

[29] Kerridge J, Kyle G, Marks-Maran D (2009). Evaluation of the use of team teaching for delivering sensitive content – a pilot study. J Furth High Educ.

[30] Anderson RS, Speck BW (1998). “Oh what a difference a team makes”: why team teaching makes a difference. Teach Teach Educ.

[31] Hatcher T, Hinton B (1996). Graduate students’ perceptions of university team teaching. Coll Stud J.

[32] Bakken L, Clark FL, Thompson J, Thompson J (1998). Collaborative teaching: many joys, some surprises, and a few Worms. Coll Teach.

[33] Griffith JW (1983). Team teaching: philosophical considerations and pragmatic consequences. J Nurs Educ.

